# Computational mass spectrometry for small molecules

**DOI:** 10.1186/1758-2946-5-12

**Published:** 2013-03-01

**Authors:** Kerstin Scheubert, Franziska Hufsky, Sebastian Böcker

**Affiliations:** 1 Chair of Bioinformatics, Friedrich Schiller University, Ernst-Abbe-Platz 2, Jena, Germany; 2 Max Planck Institute for Chemical Ecology, Beutenberg Campus, Jena, Germany

**Keywords:** Mass spectrometry, Metabolomics, Spectral library, Molecular formula identification, Structure elucidation, Fragmentation trees, Networks

## Abstract

The identification of small molecules from mass spectrometry (MS) data remains a major challenge in the interpretation of MS data. This review covers the *computational* aspects of identifying small molecules, from the identification of a compound searching a reference spectral library, to the structural elucidation of unknowns. In detail, we describe the basic principles and pitfalls of searching mass spectral reference libraries. Determining the molecular formula of the compound can serve as a basis for subsequent structural elucidation; consequently, we cover different methods for molecular formula identification, focussing on isotope pattern analysis. We then discuss automated methods to deal with mass spectra of compounds that are not present in spectral libraries, and provide an insight into *de novo* analysis of fragmentation spectra using fragmentation trees. In addition, this review shortly covers the reconstruction of metabolic networks using MS data. Finally, we list available software for different steps of the analysis pipeline.

## Introduction

Mass spectrometry (MS) is a key analytical technology for detecting and identifying small biomolecules such as metabolites [1-3]. It is orders of magnitude more sensitive than nuclear magnetic resonance (NMR). Several analytical techniques have been developed, most notably gas chromatography MS (GC-MS) and liquid chromatography MS (LC-MS). Both analytical setups have their advantages and disadvantages, see Section “Experimental setups” for details.

In recent years, it has been recognized that one of the most important aspects of small molecule MS is the automated processing of the resulting data. In this review, we will cover the development of computational methods for small molecule mass spectrometry during the last decades. Here, the term “small molecule” refers to all small biomolecules excluding peptides. Obviously, our review cannot be complete: In particular, we will not cover the “early years” of computational mass spectrometry of small molecules. First rule-based approaches for predicting fragmentation patterns, as well as explaining experimental mass spectra with the help of a molecular structure, were developed as part of the *DENDRAL* project that started back in 1965 [4-7]; see also Chapter 7 of [8]. Citing Gasteiger *et al.*[9]: “However, it is sad to say that, in the end, the *DENDRAL* project failed in its major objective of automatic structure elucidation by mass spectral data, and research was discontinued.”

We will not cover methods that deal with processing the raw data, such as de-noising and peak picking, as this is beyond the scope of our review; see Section “Software packages” for a list of available software packages for this task. Furthermore, we do not cover the problem of aligning two or more LC-MS or GC-MS runs [10-13]. Finally, we will not cover computational methods that deal with the chromatography part of the analysis, such as predicting retention indices [14,15].

Structure confirmation of an unknown organic compound is always performed with a set of independent methods, in particular NMR. The term “structure elucidation” usually refers to full *de novo* structure identification of a compound, including stereochemical assignments. It is commonly believed that structure elucidation is impossible using MS techniques alone, at least without using strong background information. We will not cover this aspect, but concentrate on the information that MS experiments *can* give.

“Computational mass spectrometry” deals with the development of computational methods for the automated analysis of MS data. Over the last two decades, much research has been focused on methods for analyzing proteomics MS data, with literally hundreds of articles being published in scientific journals [16-21]. The proteomics field has benefited tremendously from this development; often only the use of these automated methods enables high-throughput proteomics experiments. Computational methods for the analysis of proteins and peptides, as well as DNA and RNA [22,23], glycans [24-26], or synthetic polymers [27,28] are also part of computational mass spectrometry, but outside the scope of this review. Finally, disclosing methods is important for reproducible science. Thus, we will also not cover “anecdotal” computational MS where an automated method is mentioned in a paper, but no details of the method are provided.

### Review of reviews

Existing reviews on computational MS for small molecules, usually focus on a much more narrow area of the field such as raw data processing [29], metabolomics databases and laboratory information management systems [30], or metabolite identification through reference libraries [31]. Other reviews simply list available tools for processing the data without discussing the individual approaches [32].

A broad overview on experimental as well as theoretical structure elucidation techniques for small molecules using mass spectrometry is given in [33]. Methods specific for qualitative and quantitative metabolomics using LC-MS/MS are covered in [34]. Methods specific for metabolite profiling by GC-MS are covered in [35]. An overview of isotope pattern simulation is given in [36]. Annotation and identification of small molecules from fragmentation spectra using database search as well as *de novo* interpretation techniques is covered in [37].

For a general introduction to metabolomics and metabolomic profiling see [2,3,38]; for recent work in the field see [39].

### Experimental setups

Analysis of small molecules by GC-MS is usually performed using Electron Ionization (EI). Historically seen, EI is the oldest ionization technique for small-molecule investigations. Because of the selected constant ionization energy at 70 eV, resulting fragment-rich mass spectra are, in general, consistent across instruments, and specific for each compound. A major disadvantage of mass spectra obtained under EI conditions is the low abundant or missing molecular ion peak; to this end, the mass of the compound is often unknown. GC-MS requires that an analyte is volatile and thermally stable. For non-volatile analytes such as polar compounds, chemical derivatization has to be performed.

Recently, LC-MS has been increasingly used for the analysis of small molecules. Here, compounds are fragmented using tandem MS, for example by Collision Induced Dissociation (CID). This has the advantage that the mass of all molecular ions is known, which is particularly beneficial for *de novo* approaches discussed below. Unfortunately, tandem mass spectra are not as reproducible as EI spectra, in particular across different instruments or even instrument types [40]. Furthermore, using different collision energies can make tandem mass spectra hard to compare. Comparing spectra from different instrument types, only 64–89% of the spectra pairs match with more than 60*%* identity, depending on the instrument pair [41]. Finally, tandem mass spectra usually contain much less fragments than EI fragmentation spectra. Chemical derivatization can dramatically increase the sensitivity and specificity of LC-MS for less polar compounds [42].

Several methods have been proposed to create more reproducible and informative tandem MS spectra. For example, to increase the number of fragments, tandem MS spectra are often recorded at more than one fragmentation energy. Alternatively, “CID voltage ramping” continuously increases the fragmentation energy during a single acquisition [43]. Also, some progress has been made to normalize fragmentation energies across instruments and instrument types [40,44,45].

Besides the two “standard” experimental setups described above, many other setups have been developed: This includes “alternative” ionization techniques such as Matrix-Assisted Laser Desorption/Ionization [46], Atmospheric Pressure Chemical Ionization [47], Atmospheric Pressure Photoionization [48], and Desorption Electrospray Ionization [49]. Also several chromatographic methods such as High Performance LC [50] and Ultra High Performance LC (UHPLC) [51] have been developed. In particular, a sensitive capillary UHPLC shows good results in lipid identification [52]. Covering the details of these modified setups is far beyond the scope of this review. From the computational side, we can usually classify these modified setups with regards to the two “standard” setups: For example, is the mass of the molecular ion known (LC-MS/MS) or unknown (GC-EI-MS)? Is the fragmentation spectrum rich (GC-EI-MS) or sparse (LC-MS)? What is the mass accuracy of the measurement (see below)? Given that new MS technologies and experimental setups are constantly being developed, we see it as a prerequisite for a “good” method from computational MS that it is not targeted at one particular experimental setup. Note, though, that the effort required for adapting a method can differ significantly: For example, methods for identifying molecular formulas from isotope patterns (see Section “Molecular formula identification”) can be applied to any experimental setup where isotope patterns are recorded. In contrast, rule-based prediction of fragmentation spectra (see Section “In silico fragmentation spectrum prediction”) requires expert-curated “learning” of fragmentation rules.

Many methods for the computational analysis of small molecule MS, that go beyond the straightforward library search, require that masses in the mass spectra are measured with an appropriate mass accuracy. It appears that this mass accuracy is much more important for the computational analysis than the often-reported resolving power of MS instruments. Historically, GC-MS is often performed on instruments with relatively bad mass accuracy (worse than 100 ppm, parts per million). In contrast, LC-MS and tandem MS are often performed on instrumental platforms (such as Orbitrap or orthogonal Quadrupole Time-of-Flight MS) that result in a much better mass accuracy, often below 10 ppm or better. This refers to the mass accuracy that we can expect in everyday use of the instrument, not to the “anecdotal mass accuracy” of a single measurement [53]. It must be understood, though, that this is not a fundamental problem of GC-MS; in fact, GC-MS measurements of high mass accuracy are increasingly reported in the literature [54-56].

### Reporting standards for metabolomics analysis

For the maturation of metabolomics the lack of standards for presenting and exchanging data needs to be filled. MIAMET (Minimum Information About a METabolomics experiment) [57] suggests reporting standards regarding experimental design, sample preparation, metabolic profiling design and measurements. ArMet [58] is a data model that allows formal description to specify the full experimental context. The Metabolomics Standards Initiative (MSI) [59] develops guidelines and standards for sharing high-quality, structured data following the work of the proteomics community. The Data Analysis Working Group (DAWG) [60] as part of the MSI proposed reporting standards for metabolomics studies that include a reporting vocabulary and will help reproducing these studies and drawing conclusions from the resulting data. The Chemical Analysis Working Group (CAWG) established confidence levels for the identification of non-novel chemical compounds [61], ranging from level 1 for a rigorous identification based on independent measurements of authentic standards, to unidentified signals at level 4. The NIH Metabolomics Fund recently supported an initiative to create a repository that enforces the submission of metadata.

### Data storage and spectral libraries

To allow data-driven development of algorithms for small molecule identification, mass spectrometric reference datasets must be made *publicly available* via reference databases. Examples of such databases include MassBank [62,63], METLIN [64,65], Madison Metabolomics Consortium Database (MMCD) [1], Golm Metabolome Database (GMD) [66], the Platform for RIKEN Metabolomics (PRiMe) [67], or MeltDB [68]. Unfortunately, making available experimental data is much less pronounced in the metabolomics and small-molecule research community, than it is in proteomics or genomics. For example, several of the above-mentioned databases do not allow for the batch download of the database. Citing [69], “to make full use of research data, the bioscience community needs to adopt technologies and reward mechanisms that support interoperability and promote the growth of an open ‘data commoning’ culture.” Possibly, the MetaboLights database that is part of the ISA (Investigation, Study, Assay) commons framework can fill this gap. Note that the PubChem database allows free access to more than 35 million molecular structures, and this includes batch download of the data.

Besides the open (or partly open) libraries mentioned above, there exist two important commercial libraries: The National Institute of Standards and Technology (NIST) mass spectral library (version 11) contains EI spectra of more than 200 000 compounds; the Wiley Registry (9th edition) contains EI spectra of almost 600 000 unique compounds. For comparison, the GMD [66] contains EI fragmentation mass spectra of about 1 600 compounds; and the FiehnLib library contains EI spectra for more than 1 000 metabolites [70].

**Figure 1:**
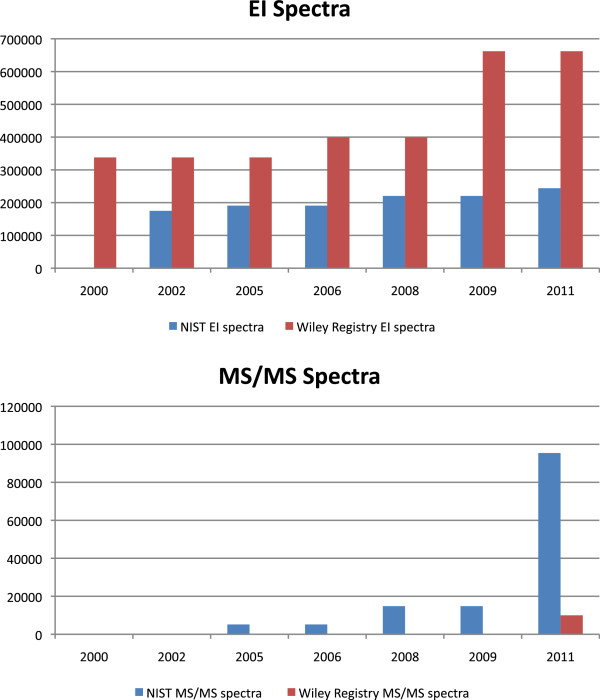
Number of EI spectra (top) and tandem mass spectra (bottom) in NIST and Wiley Registry from 2000 until 2011.

The size of tandem MS libraries is still small, compared to EI libraries (see Figure 1). The NIST 11 contains collision cell spectra for about 4 000 compounds. The Wiley Registry of Tandem Mass Spectral Data [71,72] comprises positive and negative mode spectra of more than 1 200 compounds. As for EI spectra, both databases are commercially available.

As even the commercial libraries are small, there have been several attempts to make tandem mass spectra publicly available. METLIN [64] contains high resolution tandem mass spectra for more than 10 000 metabolites for diagnostics and pharmaceutical biomarker discovery and allows to build a personalized metabolite database from its content [73]. MassBank [62,63] is a public repository with more than 30 000 spectra of about 4 000 compounds collected from different consortium members. The MMCD [1] is a hub for NMR and MS spectral data containing about 2 000 mass spectra from the literature collected under defined conditions. Some databases address specific research interests. The Human Metabolome DB [74,75] comprises reference MS-MS spectra for more than 2 500 metabolites found in the human body. The Platform for RIKEN Metabolomics (PriMe) [67,76] collects MS ^*n*^ spectra for research on plant metabolomics.

## Searching spectral libraries

The usual approach for identification of a metabolite is looking it up in a spectral library. Database search requires a similarity or distance function for spectrum matching. The most fundamental scorings are the “peak count” family of measures that basically count the number of matching peaks. A slightly more complex variant is taking the dot product of the two spectra, taking into account peak intensities.

Establishing the confidence is the more difficult part of compound identification using library search [31]. False negative identifications occur if the spectrum of the query compound differs from the spectrum in the library, for example due to contaminations, noise (especially in low signal spectra), or different collision energies (CID). A reliable identification of a compound depends on the uniqueness of its spectrum, but the presence and intensity of peaks across spectra is highly correlated, as these depend on the non-random distribution of molecular (sub-)structures. Therefore, structurally related compounds generally have similar mass spectra. Hence, false positive hits may hint at correct “class identifications”, see Section “Searching for similar compounds” below. Different from proteomics, False Discovery Rates (FDR) cannot be estimated as no appropriate decoy databases can be constructed. Usually, confidence in search results must be manually assessed by the user, based on the used search algorithm and the quality of spectrum and library [77]. Another method that overcomes this limitation is the calculation of fragmentation trees from fragmentation spectra, see Section “Fragmentation trees” below. For a review on using spectral libraries for compound identification, see [31].

### Electron ionization fragmentation spectra

To compare EI mass spectra, a huge number of scorings (or similarity measures) have been developed over the years. In 1971, the Hertz similarity index was introduced [78], representing the weighted average ratio of the two spectra. The Probability Based Matching (PBM) [79,80] takes into account that some peaks are more informative than others. Atwater *et al.*[81] statistically evaluated the effects of several parameters on the PBM system, to provide a quantitative measure of the predicted reliability of the match. *SISCOM*[82] encodes spectra by selecting the most informative peaks within homologous ion series. Computing the dot product cosine of two mass spectra (that is, the inverse cosine of the dot product of the normalized spectra) was used in the *INCOS* data system [83]. Stein and Scott [84] evaluated normalized Euclidean distances [85], PBM, Hertz similarity index, and dot product for searching EI databases. Among these, they found the dot product to perform best. They proposed a composite search algorithm that optimizes the cosine score by varying the scaling and mass weighting of the peak intensities. Koo *et al.*[86] introduced novel composite similarity measures that integrate wavelet and Fourier transform coefficients, but found only a slight improvement over cosine correlation or the composite similarity measure. Kim *et al.*[87] showed how to find optimal weight factors for fragment masses using a reference library.

Regarding the differentiation between true and bogus hits in the database, not much progress has been made: Probabilistic indicators of correct identifications using “match factors” were introduced in [88]. Jeong *et al.*[89] used an empirical Bayes model to improve the accuracy of identifications and gave a false positive estimate. For this purpose, a competition score was added to the similarity score, based on the similarity score to other spectra in the library.

### Tandem mass spectra

**Figure 2:**
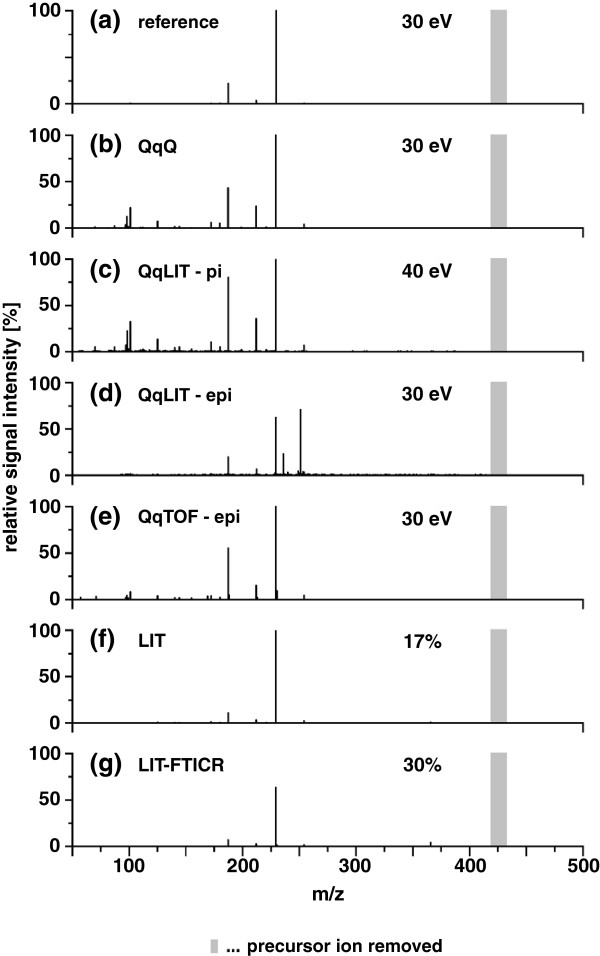
**Inter-instrument comparability of dixyrazine-specific tandem mass spectra collected on different instrumental platforms.** Figure provided by Herbert Oberacher, compare to Figure one in Oberacher *et al*[71].

We noted above that LC-MS/MS is much less reproducible than fragmentation by GC-MS (see Figure 2). Reliable library identifications can be achieved when a spectrum is acquired under the same conditions as the reference spectrum [90]. For each compound, libraries must contain tandem mass spectra at different collision energies and replicates on different instruments, to allow for an effective identification [91]. For example, Oberacher and coworkers [71,72,92] presented an inter-instrument and inter-laboratory tandem mass spectral reference library obtained using multiple fragmentation energy settings.

For searching in tandem mass spectral libraries it is possible to start with a precursor ion mass filtering with a specific m/z or mDa range. In case the actual compound is not in the database, it can be beneficial to omit this filtering step. This may reveal valuable information about structurally similar compounds [92]. Subsequently, similar approaches as for EI mass spectra can been applied, such as PBM [79,80] or dot product cosine [84,93]. Again, intensities can be weighted using peak masses [62,63]. The scoring in [92] extends the common peak count. Zhou *et al*[94] proposed a support vector machine (SVM)-based spectral matching algorithm to combine multiple similarity measures. Hansen and Smedsgaard [95] used the Jeffrey-Matusitas distance [96] to find a unique correspondence between the peaks in the two spectra.

*X-Rank* replaces peak intensities by their rank, then estimates the probability that a peak in the query spectrum matches a peak in the reference spectrum based on these ranks [97]. Oberacher *et al*[71,72] tackled the problem of low reproducibility of metabolite CID fragmentation using a dynamic intensity cut-off, counting neutral losses, and optimizing the scoring formula. To improve running times, the database can be filtered using the most intense peaks and user-defined constraints [98].

## Molecular formula identification

One of the most basic — but nevertheless highly important — steps when analyzing an unknown compound, is to determine its molecular formula, often referred to as the “elemental composition” of the compound. Common approaches first compute candidate molecular formulas using a set of potential elements. The six elements most abundant in metabolites are carbon (C), hydrogen (H), nitrogen (N), oxygen (O), phosphorus (P), and sulfur (S) [99]. For each candidate molecular formula, an isotope pattern is simulated and compared to the measured one, to determine the best matching molecular formula. For this purpose, high mass accuracy is required and is nowadays available from a multitude of MS platforms. The molecular formula of the compound can serve as a basis for subsequent structure elucidation. Some software packages for molecular formula identification using isotope patterns are summarized in Table 1.

**Table 1:** Software for the three basic steps of molecular formula identification using isotope patterns

**Decomposing monoisotopic peaks**	
*Decomp*[100,101]	for arbitrary alphabets of elements
	requires only little memory
	swift in practice
*SIRIUS*[102], [103]^∗^	implementing *Decomp* approach for MS
	decomposing real-valued masses
“Seven GoldenRules” [104]	to filter molecular formulas
**Simulating isotope patterns**	
*IsoPro*[105]	multinomial expansion to predict “center masses”
	memory- and time-consuming
*Mercury*[106]	pruning by probability thresholds and/ormass range
	reduced memory and time consumption
	reduced accuracy of the predictions
*Emass*[107]^∗^ &*SIRIUS*[102]^∗^	iterative (stepwise) computation of isotopepattern
	probability-weighted center masses
	probabilities and masses are updated as atomsare added
*IsoDalton*[108]	models the folding procedure as a Markovprocess
*BRAIN*[109]^∗^	Newton-Girard theorem and Vietes formulae to calculate intensities and masses
*Fourier*[110]^∗^	2D Fast Fourier Transform that splits up thecalculation in a coarse and a fine structure
	running time improvement for large compounds
**Scoring candidate compounds**	
*SigmaFit*	commercial software by Bruker Daltonics
*SIRIUS*[102]^∗^	Bayesian statistics for scoring intensities andmasses of the isotope pattern
*MZmine*[111]	simple scoring based only on intensities

^*^Recommended tools.

Different from the above, some authors propose to use molecular structure databases to determine the candidate molecular formulas [112]. This “simplifies” the problem as the search space is severely restricted; but only those molecular formulas can be determined where a compound is available in the structure database. To this end, we will ignore this somewhat arbitrary restriction of the search space.

In the following, we assume that elements are unlabeled or only partially labeled. If certain elements are (almost) completely labeled by heavy isotopes such as ^13^*C*, and both the unlabeled and the labeled compound are present, this allows us to directly “read” the number of atoms from the spectrum using the mass difference. We will come back to this particular type of data in Section “Isotope labeling”.

### Decomposing monoisotopic peaks

Here, “decomposing a peak” refers to finding all molecular formulas (over the fixed alphabet of elements) that are sufficiently close to the measured peak mass. Robertson and Hamming [113] and Dromey and Foyster [114] proposed a naïve search tree algorithm for this purpose. One can show that the running time of this algorithm linearly depends on *m*^*k*−1^ where *m* is the mass of the peak we want to decompose, and *k* is the number of elements [102]. This means that doubling the peak mass we want to decompose, will increase the running time of the algorithm 32-fold for the alphabet of elements CHNOPS. Hence, running time can easily get prohibitive, in particular if we consider larger alphabets of elements, or have to perform many decompositions. In 1989, Fürst *et al*[115] proposed a faster decomposition algorithm which, unfortunately, is limited to the four elements CHNO. In 2005, Böcker and Lipták [100,101] presented an algorithm that works for arbitrary alphabets of elements, requires only little memory, and is swift in practice. Initially developed for decomposing integer masses, this algorithm was later adapted to real-valued masses [102,103,116].

Decomposing alone is not sufficient to exclude enough possible molecular formulas in higher mass regions even with very high mass accuracy [117]. Kind and Fiehn [104] proposed “Seven Golden Rules” to filter molecular formulas based on chemical considerations. However, for larger masses, many molecular formulas pass these rules.

As the monoisotopic mass of a compound is insufficient to determine its molecular formula, we can use the measured isotope pattern of the compound to rank all remaining molecular formula candidates. Kind and Fiehn [117] estimated that mass spectrometers capable of 3 ppm accuracy and 2% error for isotopic abundances, can outperform mass spectrometers with hypothetical mass accuracy of 0.1 ppm that do not include isotopic information. To this end, we now consider the problems of simulating and matching isotope patterns.

### Simulating isotope patterns

Due to limited resolution of most MS instruments the isotopic variants are not fully separated in the spectra but pooled in mass bins of approximately 1 Da length. This is called the aggregated isotopic distribution [36] and in the following we will refer to it as “isotope pattern”.

Most elements have several naturally occurring isotopes. Combining elements into a molecular formula also means to combine their isotope distributions into an isotope distribution of the entire compound. Masses of all isotopes are known with very high precision [118,119]. This is, to a much lesser extend and with certain exceptions, also true for the natural abundances of these isotopes on earth [120]. (For example, the abundances of boron isotopes vary strongly.) To this end, we can simulate the theoretical isotope pattern of a molecular formula, and compare the simulated distribution to the measured pattern of a compound. See Valkenborg *et al*[36] for an introduction.

The intensity of a peak in an isotope pattern is the superposition of all isotope variants’ abundances that have identical nominal mass (nucleon number) [36]. In the early 1960’s, mass accuracy of MS instruments was relatively low. Thus, first approaches for simulating isotope patterns ignored the exact mass of the isotope peaks, and concentrate solely on isotope peak intensities, that is, the isotope distribution [121]. In 1991, Kubinyi [122] suggested a very efficient algorithm for this problem, based on convoluting isotope distributions of “hyperatoms”.

As instruments with improved mass accuracy became commercially available, focus shifted towards also predicting masses of isotope peaks, named “center masses” by Roussis and Proulx [123]. For this purpose, methods based on polynomial [124] and multinomial expansion [105,125] were developed. *IsoPro* is an implementation of [105] by M.W. Senko. Unfortunately, these expansion approaches are very memory- and time-consuming. Pruning by probability thresholds or mass range or both was introduced to reduce memory and time consumption; but this comes at the price of reduced accuracy of the predictions [106,126-128]. The approach of [106] was implemented in the software package *Mercury*.

Starting in 2004, methods that use an iterative (stepwise) computation of isotope pattern were developed [107,116,123]. These algorithms are similar in spirit to the early algorithms for computing peak intensities [121,122]. But for the new algorithms, probabilities *and masses* of isotope peaks are updated as atoms are added. This results in probability-weighted center masses. Two implementations are *Emass*[107] and *SIRIUS*[102]. To speed up computations, both approaches combine this with a smart Russian multiplication scheme, similar to Kubinyi [122].

Later approaches model the folding procedure as a Markov process [108,129,130]. *IsoDalton* implements the approach of Snider [108]. All approaches have in common that a truncation mechanism must be applied due to the exponential growth of states.

In 2012, Claesen *et al*[109] applied the Newton-Girard theorem and Vietes formulae to calculate the intensities and masses of an isotope pattern. This method is implemented in the software tool *BRAIN*. They compared their method against five other software tools: *IsoPro*, *Mercury*, *Emass*, *NeutronCluster*[131], and *IsoDalton*. In this evaluation, *BRAIN* outperformed all other software tools but *Emass* in mass accuracy of the isotope peaks. Running times were comparable for *BRAIN*, *Emass*, *Mercury*, and *NeutronCluster*, whereas *IsoPro* and *IsoDalton* required much higher computation times. Later, Böcker [132] showed that *SIRIUS* and *BRAIN* have practically identical quality of results and running times for simulating isotope patterns.

The currently fastest algorithm was presented by Fernandez-de-Cossio Diaz and Fernandez-de-Cossio [110]. This algorithm improves on earlier work were a 2D Fast Fourier Transform is applied that splits up the calculation in a coarse and a fine structure [133]. *Fourier*[110] shows a significantly better performance than *BRAIN* and, hence, *Emass* and *SIRIUS*. It must be noted, though, that this running time improvement is only relevant for large compounds: The smallest compound considered in [109,110,132] has mass above 1000 Da, and significant running time differences for *Fourier* are observed only for compounds with mass above 10 kDa. For compounds of mass above, say, 50 kDa the problem of simulating isotope patterns becomes somewhat meaningless: The abundances of isotope species are known with limited precision, and vary depending on where a sample is taken. These small deviations in the isotopic distribution of elements cause huge deviations in the aggregated distribution, if the compound is sufficiently large [134].

For the efficient and accurate simulation of isotope patterns of small compound, it is recommended to use one of the approaches behind *Fourier*[110], *BRAIN*[109], *Emass*[107], or *SIRIUS*[102].

### Scoring candidate compounds by comparing isotope patterns

**Figure 3:**
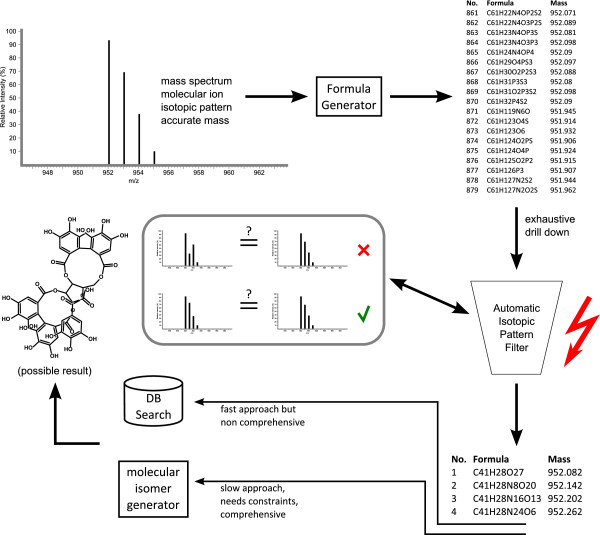
**Metabolite identification pipeline based on elemental composition calculation, isotope pattern scoring and subsequent database queries.** Figure redrawn from Kind and Fiehn [117].

Decomposing the monoisotopic peak can result in a large number of candidate molecular formulas that are within the measured mass [117]. We can rank these candidates based on evaluating their simulated isotope patterns. For each candidate molecular formula, the isotope distribution is simulated and compared with the measured one. The best matching formula is considered to be the correct molecular formula of the compound. See Figure 3.

Initially, mass spectrometers were limited in mass accuracy and resolution. To this end, first attempts of scoring isotope patterns only considered the intensity of the isotopic peaks but not their masses. Kind and Fiehn [117] calculated a root mean square error for the differences between measured and theoretical isotopic intensities. Stoll *et al*[135] filtered candidates using double-bond equivalents and number of valences, then rank candidates based on correlating the isotope distributions [136]. Commercial software for the same purpose was also provided by instrument vendors, such as *SigmaFit* by Bruker Daltonics. *Tal-Aviv*[137] targets GC-MS EI data using a supersonic molecular beam, which results in highly abundant molecular ions.

Böcker *et al*[102] introduced *SIRIUS*, first suggested in [116]. Here, both the intensities and masses of the isotope pattern are used to score candidate molecular formulas using Bayesian statistics: The authors estimate the likelihood of a particular molecular formula to produce the observed data. For a dataset of 86 compounds measured on an oa-TOF MS instrument, the correct formula was identified in more than 91% of the cases. Ipsen *et al*[138] developed a method to determine confidence regions for isotope patterns, tailored towards TOF MS data. They employ that the rate of ion arrivals at the detector plate is governed by the Poisson distribution. A test on three compounds showed that the method rejects about 70% of the candidate formulas (for pooled data) but keeps the true formula, at the 5% significance level.

### Isotope labeling

Labeling compounds by isotope-enriched elements such as ^13^*C* or ^15^*N*, helps to identify the correct molecular formula. The shift in the mass spectrum between the unlabeled compound and the labeled compound indicates the number of atoms in the compounds. Once the number of atoms for the labeled elements is known, the number of possible molecular formula is significantly reduced. Rodgers *et al*[139] showed that enrichment with 99% ^13^*C* isotopes reduces the number of possible molecular formulas for a 851 Da phospholipid from 394 to one. Hegeman *et al*[140] used isotopic labeling for metabolite identification. They improved the discriminating power by labeling with ^13^*C* and ^15^*N* isotopes. Giavalisco *et al*[141] additionally labeled compounds with ^34^*S* isotopes. By this, the number of carbon, nitrogen as well as sulfur atoms can be determined upfront, and the number of potential molecular formula that we have to consider, is reduced considerably. *Baran et al*[142] applied this approach to untargeted metabolite profiling and showed its potential to uniquely identify molecular formulas.

### Other approaches for molecular formula identification

Tandem or multiple-stage MS can give additional information about the molecular formula of the intact compound: We can exclude all molecular formulas of the compound if, for one of the fragment (product ion) peaks, we cannot find a sub-formula that explains this peak [143][146]. Unfortunately, such approaches are susceptible to noisy data. To this end, Konishi and coworkers [143][144] suggested to use only product ions below a certain threshold, e.g., 200 Da, that have a unique decomposition.

Pluskal *et al*[111] combined matching isotope patterns with filtering based on the molecular formulas of product ions. For 79% of the 48 compounds considered, they identified the correct molecular formula. There exist commercial tools that follow the same line of thought: For example, *SmartFormula3D*[146] (commercial, Bruker Daltonics) appears to implement a similar approach. Pluskal *et al*[111] also evaluated their new, simple scoring of isotope patterns against *SIRIUS*[102], and reported that it performs better.

A generalization of this concept are fragmentation trees which were initially introduced to compute molecular formulas [147]. For each potential molecular formula of the intact compound, a fragmentation tree and its score are computed. Potential molecular formulas of the compound are then sorted with respect to this score. Rasche *et al*[148] combined this with isotope pattern analysis [102], and for the 79 considered compounds measured on two instruments, they could identify the correct molecular formula in all cases. For more details on fragmentation trees, see Section “Fragmentation trees” below.

All of the above approaches assume that only the monoisotopic peak is selected for dissociation. Selecting a non-monoisotopic peak can reveal valuable information about the molecular formulas of the product ions. Singleton *et al*[149] developed an approach to predict the expected isotope pattern for tandem mass spectra for precursor ions that contain only one element with one heavy isotope. Rockwood *et al*[150] generalized this and developed an algorithm that can be applied to arbitrary precursor ions. It is based on the convolution of isotope distributions of the product ion and the loss. Again, comparing theoretical and experimental isotope patterns shed light on the correct product ion formula. Ramaley and Herrera [151] modified the algorithm from [149] to apply it to arbitrary precursor ions; results are comparable to [150].

Rogers *et al*[152] used the information of potential metabolic pathways to identify the correct molecular formula. If there is a putative chemical transformation between two molecular formulas, these formulas get a better score than other explanations of the peak. This does not only improve molecular formula identification, but can potentially be used to reconstruct biochemical networks. See Section “Network reconstruction” for details.

## Identifying the unknowns

To yield information beyond the compound mass and molecular formula, the analyte is usually fragmented, and fragmentation mass spectra are recorded. Using spectral comparison one can identify huge numbers of metabolites that are cataloged in libraries. However, where the compound is unknown, comparing the spectrum obtained to a spectral library will result in imprecise or incorrect hits, or no hits at all [33,35,99]. The limited capability for metabolite identification has been named one of the major difficulties in metabolomics [117]. Manual analysis of unidentified spectra is cumbersome and requires expert knowledge. Therefore, automated methods to deal with mass spectra of *unknown unknowns* (that is, “unexpected” compounds that are not present in spectral libraries [31]) are required. Some approaches for analyzing fragmentation mass spectra of *unknown unknowns* are summarized in Table 2.

**Table 2:** **Approaches for analyzing fragmentation mass spectra of *unknown unknowns * that is, “unexpected” compounds that are not present in spectral libraries [**[31]*]*

		***In silico* fragmentation**		
**Searching for similarcompounds**	**Mass spectral classifiers**	**Rule-based spectrumprediction**	**Combinatorialfragmentation**	**Fragmentation trees**
searching for similarspectra in a library,assuming thatspectral similarity isbased on structuralsimilarity	predicting substructures orcompound classes bylearning spectral classifiers	predicting spectra byapplying fragmentationrules to known molecularstructures	mapping the fragmentationspectrum to the compoundstructure to explainthe peaks	computing a fragmentation tree that explains the peaks; aligning fragmentation trees to find similar compounds
*NIST MSInterpreter*[153]	*FingerID*[169]	*Mass Frontier*, *ACD/MSFragmenter*, *MOLGEN-MS*[196]	*MetFrag*[179]	*SIRIUS*[147,221]

### Searching for similar compounds

In case a database does not contain the sample compound an obvious approach is to search for similar spectra, assuming that spectral similarity is based on structural similarity of the compounds. Back in 1978, Damen *et al*[82], already suggested that *SISCOM* can also be used to detect structural similarities such as common substructures.

The NIST *MS Interpreter*[153] for EI spectra uses a nearest-neighbor approach to generate substructure information. A library search provides a list of similar spectra. Structural features of the unknown compound, such as aromatic rings or carbonyl groups, are deduced from common structural features of the hits. Demuth *et al*[154] proposed a similar approach, and evaluated whether spectral similarity is correlated with structural similarity of a compound. Based on this evaluation, they proposed a threshold for spectral similarity that supposedly yields hit lists with significantly similar structures. For multiple MS data, Sheldon *et al*[155] used precursor ion fingerprints (PIF) and spectral trees for finding similar compounds and utilized previously characterized ion structures for the structural elucidation of the unknown compounds.

### Mass spectral classifiers

Another natural approach to deal with mass spectra of compounds that cannot be found in a spectral library, is to find patterns in the fragmentation spectra of reference compounds, and to use the detected patterns for the automated interpretation of the unidentified spectrum. Initially, this was accompanied by knowledge about the fragmentation processes; but this applies only for fragmentation by EI, whereas fragmentation by CID is less reproducible and not completely understood [156].

To characterize an unknown compound, we have to come up with “classifiers” that assign the unknown to a certain class: such classes can be based on the presence or absence of certain substructures, or more general structural properties of the compound. As EI fragmentation is already well understood, many mass spectral classifiers have been provided to date. Already in 1969, Venkataraghavan *et al*[157] presented an automated approach “to identify the general nature of the compound and its functional groups.” The Self-Training Interpretive and Retrieval System (*STIRS*) [158] mixes a rule-based approach with some early machine learning techniques to obtain structural information from related EI spectra. Further, *STIRS* can predict the nominal molecular mass of an unknown compound, even if the molecular ion peak is missing from the EI spectrum. Scott and coworkers [159,161] proposed an improved method for estimating the nominal molecular mass of a compound. Using pattern recognition the compound is classified, and class-specific rules are applied to estimate the molecular mass.

Structural descriptors (that is, fragments of a certain integral mass) have been used to retrieve compound classes for many decades [162]. The Varmuza feature-based classification approach for EI spectra [163] uses a set of mass spectral classifiers to recognize the presence/absence of 70 substructures and structural properties in the compound. This approach is integrated to *MOLGEN-MS* and *AMDIS*. For example, Schymanski *et al*[164] combined mass spectral classifiers with methods for structure generation (see Section “Molecular isomer generators”) to interpret EI spectra classifiers from *MOLGEN-MS* and the NIST05 software. Further MS classifiers for substructures are provided in [165,166]. Hummel *et al*[167] used structural features to subdivide the Golm Metabolome Database into several classes. They proposed a decision tree-based prediction of the most frequent substructures, based on mass spectral features and retention index information, for classification of unknown metabolites into different compound classes. In 2011, Tsugawa *et al*[168] used Soft Independent Modeling of Class Analogy (SIMCA) to build multiple class models. However, back in 1996, Varmuza and Werther [163] observed that SIMCA (which is based on the Principle Component Analysis) performed worst among all investigated methods.

**Figure 4:**
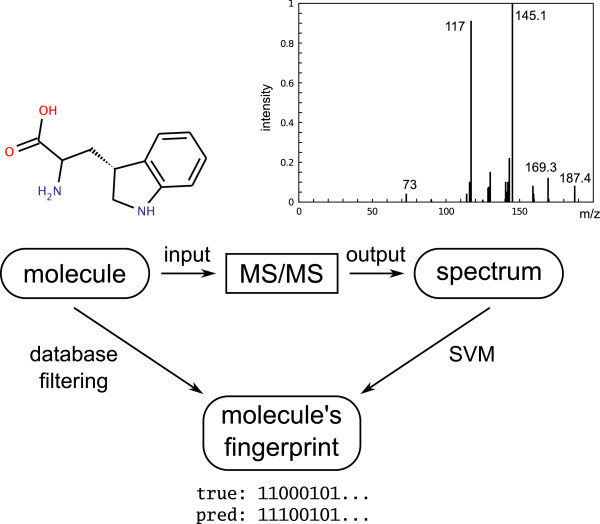
**Predicting chemical properties (molecule fingerprints) from tandem MS data using a support vector machine (SVM) as done by Heinonen*et al***[169]**.** The predicted fingerprints are used to search a molecular structure database for metabolite identification. Figure redrawn from Heinonen *et al*[169].

Whereas all of the above methods are targeted towards GC-MS and EI fragmentation, few methods target LC-MS and CID fragmentation. A novel approach by Heinonen *et al*[169] predicts molecular properties of the unknown metabolite from the mass spectrum using a support vector machine, then uses these predicted properties for matching against molecular structure databases such as KEGG (Kyoto Encyclopedia of Genes and Genomes) and PubChem (see Figure 4). To this end, we can replace the small spectra libraries by the much larger structure databases. Using QqQ MS data and searching the smaller KEGG database, they could identify the correct molecular structure in about 65% of the cases, from an average of 25 candidates.

### Molecular isomer generators

Molecular isomer generators such as *MOLGEN*[170,172], *SMOG*[173], and *Assemble*[174] have helped with the structural elucidation of unknowns for many years [175,176]. Recently, the open source software OMG was introduced [177]. Molecular isomer generators enumerate all molecular structures that are chemically sound, for a given molecular formula or mass. In addition, the space of generated structures can be constrained by the presence or absence of certain substructures, see Section “Mass spectral classifiers”. An overview on generating structural formulas is given by Kerber *et al*[172]. Enumerating all possible isomers allows us to overcome the boundaries of database searching: Simply generate all molecular structures corresponding to the parent mass or molecular formula, and use the output of the structure generator as a “private database”. Unfortunately, this approach is only valid for relatively small compounds (say, up to 100 Da): For molecular formula *C*8*H*6*N*2*O* with mass 146 Da there exist 109 240 025 different molecular structures [172].

### In silico fragmentation spectrum prediction

*In silico* fragmentation aims to explain “what you see” in a fragmentation spectrum of a metabolite. Initially, this was targeted at a manual interpretation of fragmentation spectra; but recently, this approach has been increasingly used for an automated analysis [178,179]. Here, searching in spectral libraries is replaced by searching in molecular structure databases. We mentioned above that spectral libraries are (and will be) several orders of magnitude smaller than molecular structure databases: For example, the CAS Registry of the American Chemical Society and PubChem currently contain about 25 million compounds each. We can also use molecular structure generators (see “Molecular isomer generators”) to create a “private database”. However, whereas structure generators can enumerate millions of structures in a matter of seconds, it is already a hard problem to rank the tens or hundreds of molecular structures found in molecular structure databases for a particular parent mass [178,179].

*In silico* fragmentation has been successfully applied to compounds with consistent fragmentation pattern, such as lipids [180], oligosaccharides [181], glycans [182], peptides [183,185] or non-cyclic alkanes and alkenes [186]. However, general fragmentation prediction of arbitrary small molecule remains an active field of research, due to the structural diversity of metabolites and the complexity of their fragmentation patterns.

Basically there are two types of *in silico* fragmentation methods. *Rule-based fragmenters* are based on fragmentation rules that were extracted from the MS literature over the years. *Combinatorial fragmenters* use a bond disconnection approach to dissect a compound into hypothetical fragments.

#### *Rule-based fragmenters*

Although much is known about EI fragmentation, it is a hard ionization technique that can result in very complex rearrangements and fragmentation events [187] which are hard to predict. For tandem MS, the fragmentation behavior of small molecules under varying fragmentation energies is not completely understood [156], and has been investigated in many studies to find general fragmentation rules [188,189]. *Mass Frontier* (see below) currently contains the largest fragmentation library, manually curated from several thousand publications [33].

The first rule-based approaches for predicting fragmentation patterns and explaining experimental mass spectra with the help of a molecular structure were developed as part of the *DENDRAL* project. For example, Gray *et al*[190] introduced *CONGEN* that predicts mass spectra of given molecular structures using general models of fragmentation, as well as class-specific fragmentation rules. Intensities for EI spectra were modeled with equations found by multiple linear regression analysis of experimental spectra and molecular descriptors [191].

*Gasteiger et al*[9] introduced *MASSIMO* (MAss Spectra SIMulatOr) to automatically derive knowledge about mass spectral reaction types directly from experimental mass spectra. Part of *MASSIMO* is the Fragmentation and Rearrangement ANalyZer (*FRANZ*) that requires a set of structure-spectrum-pairs as input. The MAss Spectrum SImulation System (*MASSIS*) [192,194] combines cleavage knowledge (McLafferty rearrangement, retro-Diels-Alder reaction, neutral losses, oxygen migration), functional groups, small fragments (end-point and pseudo end-point fragments) and fragment-intensity relationships for simulating electron ionization spectra. Unfortunately, these three software packages were neither sufficiently validated nor made publicly available. As a consequence, they were never used or applied by the broad community and should be considered with caution.

*Mass Frontier* (HighChem, Ltd. Bratislava, Slovakia; versions after 5.0 available from Thermo Scientific, Waltham, USA) contains fragmentation reactions collected from mass spectrometry literature. Besides predicting a spectrum from a molecular structure, it can also explain a measured fragmentation spectrum. The *ACD/MS Fragmenter* (Advanced Chemistry Labs, Toronto, Canada) can only interpret a given fragmentation spectrum using a known molecular structure [195]. Initially, these programs were designed for the prediction and interpretation of fragmentation by EI, but recently, there has been a tendency to interpret tandem MS data with theses programs, too. Both programs are commercial, and no algorithmic details have been published. A third commercial tool is *MOLGEN-MS*[196,197] that uses general mass spectral fragmentation rules but can also accept additional fragmentation mechanisms.

For the interpretation of tandem mass spectra, Hill *et al*[178] proposed a “rule-based identification pipeline”. First, they retrieved candidate molecular structures from PubChem using exact mass. Next, *Mass Frontier* 4 was used to predict the tandem mass spectra of the candidates, which were matched to the measured spectrum, counting the number of common peaks. In this way, a rule-based fragmenter can be used to search in a molecular structure database. Pelander *et al*[198] used *ACD/MS Fragmenter* for drug metabolite screening by tandem MS. For the simulation of EI fragmentation spectra, Schymanski *et al*[195] compared the three commercial programs, and indicated that at the time of evaluation, mass spectral fragment prediction for structure elucidation was still far from daily practical usability. The authors also noted that *ACD Fragmenter* “should be used with caution to assess proposed structures [ …] as the ranking results are very close to that of a random number generator.” Later, Kumari *et al*[199] implemented a pipeline for EI spectra integrating *Mass Frontier* that is similar to the one for tandem MS data [178], but integrates retention time prediction. They retrieved candidate structures from PubChem using molecular formulas predicted from the isotope pattern [104]. They filtered molecular structures using Kováts retention index prediction [15]. Using *Mass Frontier* 6 for spectrum prediction, the correct structure was reported in 73% within the TOP 5 hits.

It is worth mentioning that rule-based systems did not have much success in proteomics: There, it is apparent from the very beginning that, in view of the huge search space, only optimization- and combinatorics-based methods can be successful.

#### *Combinatorial Fragmenters*

The problem with rule-based fragmenters is that even the best commercial systems cover only a tiny part of the rules that should be known. Constantly, new rules are discovered that have to be added to the fragmentation rule databases. However, all of these rules do not necessarily apply to a newly discovered compound.

**Figure 5:**
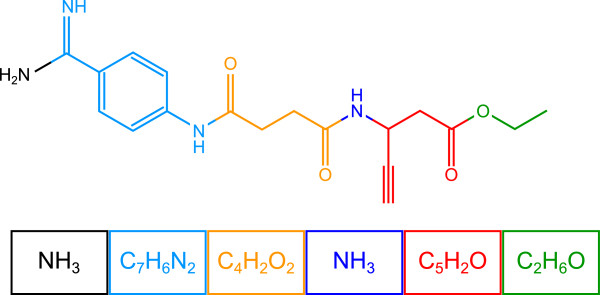
**Modular structure of xemilofiban.** Figure redrawn from Sweeney [200].

Sweeney [200] observed that many compounds can be described in a modular format, that is, substructures which account for most of the fragments observed in the fragmentation spectrum (see Figure 5). Combinatorial fragmenters use bond disconnection to explain the peaks in the observed fragmentation spectrum. Fragments resulting from structural rearrangements are initially not covered by this approach. Usually, such rearrangements have to be individually “woven” into the combinatorial optimization; this is often complicated and done only for a few, particularly important rearrangements. Note that handling rearrangement reactions is problematic for both combinatorial and rule-based methods [200,202].

*EPIC* (elucidation of product ion connectivity) [201] was the first software using systematic bond disconnection and ranking of the resulting substructures. It was tested only against two hand annotated spectra from the literature and is not publicly available. The Fragment iDentificator (*FiD*) [202,203] enumerates all possible fragment candidates using a Mixed Integer Linear Programming approach, and ranks the candidates according the cost of cleaving a fragment. Due to the computational complexity of the underlying problem [204], running times can be prohibitive even for medium-size compounds.

**Figure 6:**
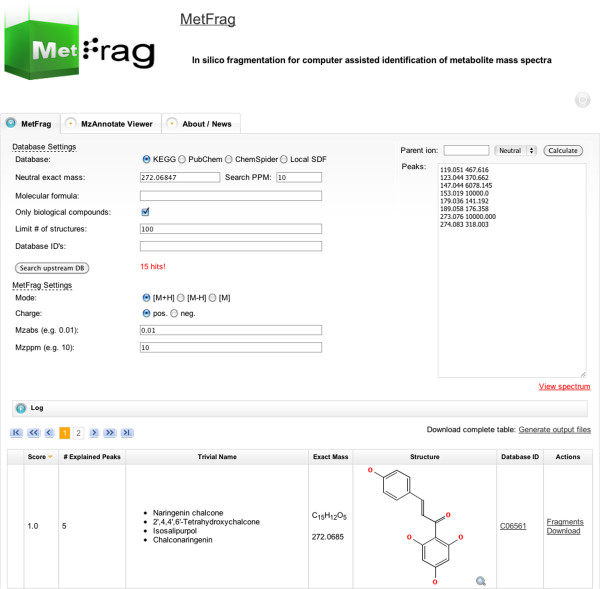
**MetFrag web interface with an example spectrum from Naringenin.** Searching KEGG as compound library with an 10 ppm window returns 15 hits, and the correct molecule is ranked at first position.

The most recent approach is *MetFrag*[179], a somewhat greedy heuristic to match molecular structures to measured spectra that makes no attempt to create a mechanistically correct prediction of the fragmentation processes. It is therefore fast enough to screen dozens to thousands of candidates retrieved from compound databases, and to subsequently rank them by the agreement between measured and *in silico* fragments (see Figure 6). Hill *et al* On the same test set that was used by [178], *MetFrag* performed better than the commercial *Mass Frontier* 4. *MetFrag* predictions were included in the recent METLIN database release [65]. *MetFrag* has also been extended to analyze EI fragmentation [205]. Recently, Gerlich and Neumann [206] introduced *MetFusion* that combines the *MetFrag* approach with a similarity fingerprint to re-rank the molecular structures.

Other experimental measures such as retention indices or drift time, can be used for candidate filtering [205,207]. *Ridder et al*[208] presented a closely related approach for substructure prediction using multistage MS data.

One problem of combinatorial fragmenters is how to choose the costs for cleaving edges (bonds) in the molecular structure graph. For this, *MetFrag* uses bond dissociation energies whereas “unit weights” are used in [208]. Kangas *et al*[180] used machine learning to find bond cleavage rates. Their *In silico* identification software (*ISIS*) currently works only for lipids and is not modeling rearrangements of atoms and bonds. Different from the other approaches, *ISIS* simulates the spectrum of a given lipid, and does not require experimental data to do so.

### Consensus structure approaches

Many of the above mentioned techniques are rather complementary yielding different information on the unknown compound. Combining the different results will therefore greatly improve the identification rates. For EI fragmentation data, [205] used a consensus scoring to selected candidates. These structural candidates are generated using molecular formula and substructure information retrieved from *MOLGEN-MS* and *MetFrag*, and further characteristics (e.g., retention behavior). Ludwig *et al*[209] proposed a greedy heuristic to find the characteristic substructure that is “embodied” in a list of database search results; see also Section “Fragmentation trees”.

### Nonribosomal peptides

Usually the structure of small molecules cannot be deduced from the genomic sequence. However, for particular molecules such as nonribosomal peptides (NRPs) a certain predictability has been established [210]. NRPs are excellent lead compounds for the development of novel pharmaceutical agents such as antibiotics, immunosuppressors, or antiviral and antitumor agents [211]. They differ from ribosomal peptides in that they can have a non-linear structures (for example, cyclic or tree-like) and may contain non-standard amino acids [211]. This increases the number of possible building blocks from 20 to several hundreds, and certain amino acid masses not even known in advance. To this end, common approaches for sequencing ribosomal peptides using tandem mass spectrometry are not applicable to NRPs. For cyclic peptides, fragmentation steps beyond tandem MS are required, as tandem MS simply results in the linearization of the cyclic peptide. Nevertheless, NRPs are structurally much more restricted than the vast variety of metabolites known from plants or microbes. Computational methods for *de novo* sequencing and dereplication of NRPs have been established [17,211,214]. Unfortunately, these computational methods rely on the “polymeric character” of NRPs and, hence, cannot be generalized for analyzing other classes of metabolites.

## Fragmentation trees

If we want to assign molecular formulas to the precursor and product ions, we may use the formula of the precursor to filter bogus explanations of the product ions, and *vice versa*. This fact has been exploited repeatedly, see for example [111,146] and Section “Molecular formula identification” above. This is only the most simplistic description of the fragmentation process: It is obvious that all product ions must be fragments of the precursor; but what is the dependency between the fragments? In fact, MS experts have drawn fragmentation diagrams for decades. For this task, the MS expert usually has to know the molecular structure of the compound and its tandem MS fragmentation spectrum.

Fragmentation trees must not be confused with *spectral trees* for multiple stage mass spectrometry [155], or the closely related *multistage mass spectral trees* of Rojas-Cherto *et al*[145] (referred to as “fragmentation trees” in [145,215,216]). Spectral trees are a formal representation of the MS setup and describe the relationship between the MS ^*n*^ spectra, but do not contain any additional information. We stress that all computational approaches described below target *tandem MS*, unless explicitly stated otherwise. To compute a fragmentation tree, we need neither spectral libraries nor molecular structure databases; this implies that this approach can target “true unknowns” that are not contained in *any* molecular structure database.

**Figure 7:**
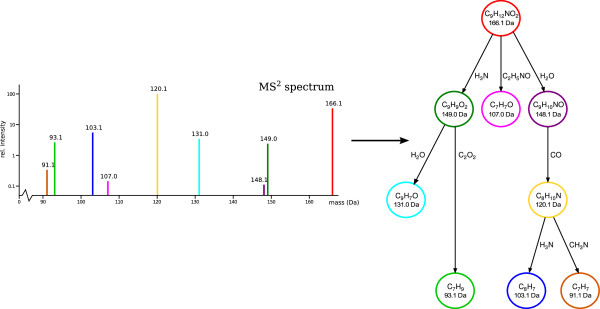
Fragmentation tree of phenylalanine computed from tandem MS data.

Böcker and Rasche [147] introduced fragmentation trees (see Figure 7) to find the molecular formula of an unknown, without using databases: Here, the highest-scoring fragmentation tree for each molecular formula candidate is used as the score of the molecular formula itself. Only later, fragmentation trees were conceived as a means of structural elucidation [148]. Algorithmic aspects of computing fragmentation trees were considered in [217]. Hufsky *et al*[56] computed fragmentation trees from EI fragmentation spectra with high mass accuracy, and used this to identify the molecular ion peak and the molecular formula of compounds. Fragmentation trees computed from both tandem MS [148] and EI fragmentation data [218] were found to be of good “structural quality” by expert evaluation. Finally, Scheubert *et al*[219,220] computed fragmentation trees from multiple MS data.

To further process fragmentation trees, Rasche *et al*[221] introduced fragmentation tree alignments to cluster unknown compounds, to predict chemical similarity, and to find structurally similar compounds in a spectral library using *FT-BLAST* (Fragmentation Tree Basic Local Alignment Search Tool). *FT-BLAST* also offers the possibility to identify bogus hits using a decoy database, allowing the user to report results for a pre-defined False Discovery Rate. Faster algorithms for the computationally demanding alignment of fragmentation trees were presented in [222]. *FT-BLAST* results were parsed for “characteristic substructures” in [209]. Rojas-Chertó *et al*[215] presented a related approach for the comparison of multistage mass spectral trees, based on transforming the trees into binary fingerprints and =comparing these fingerprints using the Tanimoto score (Jaccard index). This was applied for metabolite identification in [216].

Aligning fragmentation trees is similar in spirit to the feature tree comparison of Rarey and Dixon [223]. Feature trees were computed from the molecular structure of a *known* compound, and represent hydrophobic fragments and functional groups of the compound, and the way these groups are linked together.

## Network reconstruction

Network elucidation based on mass spectrometry data is a wide field. On the one hand, detailed information like quantitative fluxes of the network is achieved by metabolic flux analysis. Here, based on isotope labeled compounds, the flux proceeding from these compounds can be tracked. On the other hand, measured metabolites can be mapped on a known network. This can elucidate distinct metabolic pathways that are differentially “used” dependent on environmental conditions. Both of these variants require previous known metabolic network graphs. In this section, we will only cover the pure *de novo* reconstruction of networks from metabolite mass spectrometry data.

The reconstruction of networks solely from metabolic mass spectrometry data is a very young field of research. It can be subdivided into two main approaches: either the network reconstruction is based on metabolite level correlation of multiple mutant and wild type samples, or on data from only one sample by using information of common reactions or similarity between metabolites.

A first approach that used metabolite mass spectrometry data of multiple expressed samples was introduced by Fiehn *et al*[224]. Their method clusters metabolic phenotypes for example by principle component analysis (PCA). In contrast Arkin *et al*[225] and Kose *et al*[226] developed a method that does not group samples but metabolites with correlating intensity regarding all samples. Metabolites of a group have a similar concentration behavior in all samples. This leads to the assumption that the metabolites of a group are probably somehow connected in a metabolic network. As the concentration of metabolites taken from plants with identical genotype and grown under uniform conditions still show variability, this approach can also be used if no multiple mutant genotypes are available [227]. The disadvantage of this simple approach is, that it results in very dense networks that do not only cover direct reactions but also indirect ones. Krumsiek *et al*. 2011 [228] suggested to apply Gaussian graphical models to such data. Gaussian graphical networks have the ability to calculate only direct correlations while indirect correlations are not taken into account.

In 2006, Breitling *et al*[229] reconstructed networks based on high-resolution mass spectrometry data of only one dataset. They inferred accurate mass differences between all measured metabolites. These mass differences give evidences of biochemical transformations between the metabolites and allow the reconstruction of a network. Rogers *et al*[152] used a similar approach on molecular formula level to assign better molecular formulas to metabolites (see Section “Other approaches for molecular formula identification”).

**Figure 8:**
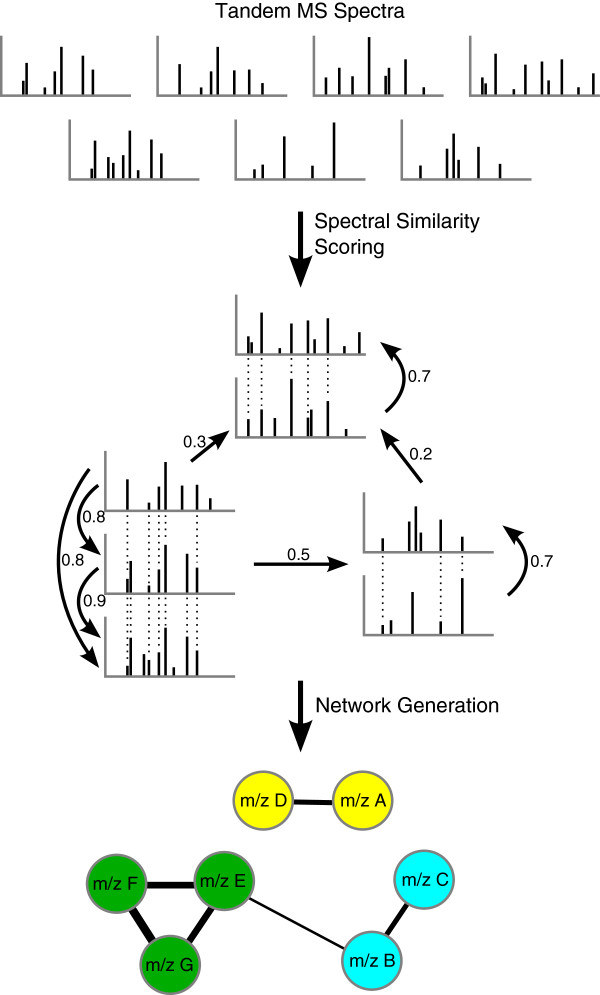
**Using spectral alignment of tandem MS data to generate a molecular network.** The thickness of the edges indicates the similarity between the spectra. Figure redrawn from Watrous *et al*[230].

Watrous *et al*[230] used additional information from spectral alignments of tandem MS data to determine a structural similarity between the metabolites. Two structurally similar metabolites are supposed to be connected in the network (see Figure 8). They found the compound thanamycin in *Pseudomonas sp. SH-C52* that has an antifungal effect and protects sugar beet plants from infections by specific soil-borne fungi.

## Software packages

Several open source, or at least freely available, software packages assist with processing and analyzing GC-MS metabolomics data. The freely available *AMDIS*[231] is the most widely used method for extracting individual component spectra (mass spectral deconvolution) from GC-MS data. *MathDAMP*[232] helps with the identification and visualization of differences between complex metabolite profiles. *TagFinder*[233,234] supports the quantitative analysis of GC-MS-based metabolite profiling experiments. The *MetaboliteDetector*[235] detects and subsequently identifies metabolites and allows for the analysis of high-throughput data. *TargetSearch*[236] iteratively corrects and updates retention time indices for searching and identifying metabolites. *Metab*[237] is an R package that automates the pipeline for analysis of metabolomics GC-MS datasets processed by *AMDIS*. *PyMS*[238] comprises several functions for processing raw GC-MS data, such as noise smoothing, baseline correction, peak detection, peak deconvolution, peak integration, and peak alignment. *ADAP-GC* 2.0 [239] helps with the deconvolution of coeluting metabolites, aligns components across samples and exports their qualitative and quantitative information. Castillo *et al*. 2011 [240] developed a tool to process GC ×GC-TOF-MS data.

For LC-MS data, *XCMS*[13] enables retention time alignment, peak detection and peak matching. *XCMS*^2^[241] additionally searches LC-MS/MS data against METLIN and also provides structural information for unknown metabolites. It also allows for the correction of mass calibration gaps [242] caused by regular switches between the analyte and a standard reference compound. *XCMS Online*[243] is the web-based version of the software. *AStream*[244] enables the detection of outliers and redundant peaks by intensity correlation and retention time, as well as isotope detection. *MetSign*[245] provides several bioinformatics tools for raw data deconvolution, metabolite putative assignment, peak list alignment, normalization, statistical significance tests, unsupervised pattern recognition, and time course analysis. *CAMERA*[246] is designed to post-process *XCMS* feature lists and integrates algorithms to extract compound spectra, annotate peaks, and propose compound masses in complex data. *MetExtract*[247] detects peaks corresponding to metabolites by chromatographic characteristics and isotope labeling. *IDEOM*[248] filters and detects peaks based on *XCMS*[13] and *mzMatch.R*[249], enables noise filtering based on [249,250] and allows for database matching and further statistics. Brodsky *et al*[251] presented a method for evaluating individual peaks in a LC-MS spectrum, based on replicate samples.

For both, GC-MS and LC-MS data, *MZmine*[252] and *MZmine2*[253] allow for data visualization, peak identification and peak list alignment. *MET-IDEA*[254] proceeds from complex raw data files to a complete data matrix. *MetAlign*[255] is capable of baseline correction, peak picking, as well as spectral alignment.

To compare the power of these software packages, an independent validation would be desirable. But up to now, there exists no such comparison. One reason is the limited amount of freely available mass spectra, see Section “Conclusion”. Another reason is that some of the packages are developed for special experimental setups or instruments, and have to be adapted for other data, what makes an independent validation difficult.

## Conclusion

No computational *de novo* method is able to elucidate the structure of a metabolite solely from mass spectral data. They can only reduce the search space or give hint to the structure or class of the compound. Computational mass spectrometry of small molecules is, at least compared to proteomics, still very much in a developmental state. This may be surprising, as methods development started out many years before computational mass spectrometry for proteins and peptides came into the focus of bioinformatics and cheminformatics research [183,185]. But since then, methods development in computational proteomics has proliferated [16,21] and long surpassed that in metabolomics and small molecule research. To a great extend, this can be attributed to the fact that freely sharing data and benchmark test sets has become a tradition in proteomics, providing developers of novel computational methods with the required input for training and evaluation of their methods.

In metabolomics, a comparative evaluation of methods is very limited due to restricted data sharing. Recently, a first benchmark test for small molecules was provided as part of the CASMI challenge^a^. CASMI is a contest in which GC-MS and LC-MS data is released to the public, and the computational mass spectrometry community is invited to identify the compounds. Results and methods will be published in a special issue of the Open Access MDPI journal *Metabolites*. This is a first step towards reliable evaluation of different computational methods for the identification of small molecules. Lately, the importance of computational methods has gained more attention in small molecule research: Citing Kind and Fiehn [33], “the ultimate success of structure elucidation of small molecules lies in better software programs and the development of sophisticated tools for data evaluation.”

With the advent of novel computational approaches [169,206,207], searching spectral libraries may be replaced by searching molecular structure databases within in the next five to ten years. Beyond molecular databases, only few approaches aim at overcoming the limits of the “known universe of organic chemistry” [256], one example being fragmentation trees [56,148,221].

## Endnote

^a^Critical Assesment of Small Molecule Identification, http://casmi-contest.org/.

## Competing interests

The authors declare that they have no competing interests.

## Authors’ contributions

KS wrote the Sections “Molecular formula identification” and “Network reconstruction”. FH wrote the Sections “Searching spectral libraries” and “Identifying the unknowns”. SB wrote the Section “Fragmentation trees”. All authors read and approved the final manuscript.
